# CD154 Restricts Helminth‐Induced Macrophage Polarisation and Proliferation While Promoting Tissue Residence

**DOI:** 10.1111/pim.70043

**Published:** 2025-12-12

**Authors:** Mariana Suárez‐Martins, James Parkinson, Lili Zhang, Brian Chan, Georgia Baldwin, Rebecca J. Dodd, Conor M. Finlay, Pedro Papotto, Judith E. Allen, Álvaro Díaz

**Affiliations:** ^1^ Área Inmunología ‐ Departamento de Biociencias Facultad de Química ‐ Instituto de Higiene, Universidad de la República Montevideo Uruguay; ^2^ Lydia Becker Institute of Immunology and Inflammation, School of Biological Sciences, Faculty of Biology Medicine and Health, University of Manchester Manchester UK; ^3^ Trinity Kidney Centre Trinity Translational Medicine Institute, School of Medicine, Trinity College Dublin Dublin Ireland

**Keywords:** cell differentiation, cell proliferation, helminths, interleukin‐4, macrophages, peritoneal cavity, T‐lymphocytes

## Abstract

CD154 (CD40L) is of central importance in effector responses mediated by classically activated macrophages. However, there is limited information on the impact of CD154 on macrophage responses in type 2 contexts, characterised by IL‐4‐induced polarisation and proliferation. CD154 restricts the polarisation and proliferation of peritoneal cavity macrophages in response to exogenous IL‐4. Here we address the impact of CD154 on peritoneal macrophages during infection of mice with the intestinal helminth *Heligmosomoides polygyrus.* This strictly enteric nematode causes recruitment of Th2 cells and macrophages to the peritoneal cavity alongside type 2 polarisation and proliferation of recruited and resident macrophages. Nine days post‐infection, there was an increase in the expression of cell‐surface CD154 in CD4^+^ T cells together with increased IL‐13 levels in the cavity, suggestive of local antigen presentation. Blocking CD154 enhanced the proliferation of resident but not of recently recruited macrophages. CD154 blocking additionally potentiated the expression of type 2 marker Ym1 (*Chil3*) in resident and recruited macrophages. Unexpectedly, CD154 blocking caused increases in the numbers of recently recruited macrophages and the appearance of cells with characteristics of both differentiating recruited macrophages (expression of folate receptor β and MHCII) and resident macrophages (high‐level expression of F4/80 and CD102). Together, these observations suggest that CD154 promotes the acquisition of tissue residence by recruited macrophages. Thus, our results indicate that in a helminth infection CD154 restricts certain aspects of the polarisation and proliferation of macrophages in response to type 2 cytokines while promoting the acquisition of resident phenotype by the recruited macrophages.

## 1 Introduction

CD154 (CD40 ligand, CD40L) is a transmembrane protein from the tumour necrosis factor (TNF) family that is up‐regulated in CD4^+^ T cells activated by antigen‐specific interactions [1, 2, 3, 4, 5]. T‐cell receptor (TCR) engagement triggers transcription from the CD154 gene, and antigen‐experienced CD4^+^ T cells have intracellular stores of CD154 protein [6, 7, 8, 9, 10, 11, 12]. TCR signalling additionally causes externalisation of CD154, both in memory and in newly activated CD4^+^ T cells [7, 11, 12, 13]. In addition, naïve CD4^+^ T cells constitutively express modest levels of CD154, mainly found at the cell surface [11, 14, 15]. The most important receptor for CD154 is CD40, a TNF receptor family protein expressed in antigen‐presenting cells (APCs) [1, 2, 3, 4]. CD40 engagement activates APCs for enhanced expression of major histocompatibility complex II (MHCII), costimulatory molecules, and cytokines that in turn signal to T cells. Thus the CD154‐CD40 interaction is a central player in the intercellular dialogues between CD4^+^ T cells and DCs, B cells and macrophages [1, 3, 4, 5, 16, 17, 18, 19, 20, 21, 22, 23, 24].

CD154 promotes the classical activation of macrophages, including expression of IL‐12 [16, 17, 18, 19, 20]. It is thus conceivable that CD154 should oppose the effect of the type 2 cytokines IL‐4 and IL‐13 on macrophage polarisation and proliferation [25, 26, 27]. However, reality could be more complex as CD154 and other CD40 agonists can have different effects depending on dose, format (soluble vs. cell‐surface) and APC subtype involved [28, 29, 30]. We recently showed that antigen‐induced CD154 or high‐dose soluble CD40 agonists can indeed restrict macrophage polarisation and proliferation in response to exogenous IL‐4, using basal and thioglycollate‐recruited peritoneal cavity macrophages as model systems [15]. Here we extend that analysis to *Heligmosomoides polygyrus* infection in mice [25, 31]. In this model, larvae infect the host orally and 24 h later invade the duodenal wall, where they develop into adult worms that return to the gut lumen starting from day 8 post‐infection [32, 33]. In spite of this being a strictly enteric infection, it causes Th2 cells and macrophages to be recruited to the peritoneal cavity and macrophages (recruited and resident) at the site to polarise and proliferate in response to type 2 cytokines [26, 34, 35, 36, 37, 38, 39]. Our results show that *H. polygyrus* infection induces the upregulation of CD154 in CD4^+^ T cells in the peritoneal cavity and blocking CD154 enhances the local proliferation and type 2 (M(IL‐4)) polarisation of macrophages. In addition, our results suggest that blocking CD154 delays the acquisition of residency by macrophages recruited in response to infection.

## 2 Materials and Methods

### 2.1 Parasite, Mice, Infections and CD40L Blocking

Female C57BL/6 mice were kept at the University of Manchester Biological Services and fed normal chow and water *ad libitum*. After being randomly assigned to cages, mice were either infected by oral gavage with 200 *H. polygyrus* L3 (kindly provided by Dr. John Grainger, from the Division of Immunology, Immunity to Infection and Respiratory Medicine, University of Manchester) or untreated. Six days later, mice were injected i.p. with 100 μg of the CD154 blocking (clone: MR1) antibody or hamster IgG as a control (both from Biolegend). Mice were euthanized on day 9 post‐infection for the analysis of peritoneal cells, retrieval of mesenteric lymph nodes, and quantification of worm burden in the intestine. For this purpose, intestines were opened longitudinally, wrapped in gauze and incubated overnight at 37°C in tubes containing PBS. Worms were then recovered from the bottom of the tubes, incubated for 2 h at 56°C to facilitate their separation, fixed with formalin, and counted under a stereomicroscope. When used, EdU (0.5 mg per mouse) was injected i.p. 16 h before euthanasia. Experiments were performed in accordance with the UK Animals Act (1986) under a Project Licence granted by the UK Home Office and approved by the University of Manchester Animal Welfare and Ethical Review body (PP4115856).

### 2.2 Flow Cytometry

Cells were stained as detailed in [15] using the flow cytometry probes listed in Table S1. Data were acquired in a LSRFortessa X‐20 (BD Biosciences) and analysed using FlowJo version 10.10.0. CD154^+^ cells were discriminated using a FMO control as a threshold. Gating strategies are presented in Figure S1.

### 2.3 Intracellular Cytokine Measurements in CD4^+^ T Cells

Mesenteric lymph node cells were plated in 96‐well U‐bottom plates, stimulated for 4 h with eBioscience Cell Stimulation Cocktail (plus protein transport inhibitors) (ThermoFisher), and stained for the intracellular cytokines.

### 2.4 Quantification of IL‐13

Soluble peritoneal cavity contents, retrieved by peritoneal lavage with 1 mL of RPMI medium with 0.2% w/v fetal bovine serum, were analysed by enzyme‐linked immunosorbent assay (ELISA) for the quantification of IL‐13. The eBioscience rat monoclonals eBiol3A and biotinylated Bio1316H were used for capture and detection respectively, followed by streptavidin‐peroxidase from KPL, and IL‐13 standard from PeproTech.

### 2.5 Uniform Manifold Approximation and Projection (UMAP) Visualisation

These were carried out using the FlowJo software with the UMAP v2.1 plugin and applied to live CD19^−^ SiglecF^−^ Ly6G^−^ CD11b^+^ cells. The data from each experiment were concatenated and used as input for building the UMAPs. Input variables used were forward scatter, side scatter, and fluorescence intensities for CD11b, F4/80, MHCII, folate receptor β (FRβ), CD73, and CD102. Cell clusters were defined on the basis of the gating strategy shown in Figure S1 and also in an unsupervised fashion using the FlowSOM plugin in FlowJo.

### 2.6 Statistical Analysis

Most of the data, corresponding to repeated experiments, were analysed by robust two‐way parametric statistics in the R software environment, as detailed in [15]. Two‐way methods allow the detection of differences between experimental groups that are consistent across repeated experiments even when absolute values (for each group) differ among experiments. In a case in which repeated experiments were not available, data were analysed by one‐way ANOVA in the GraphPad Prism package. False discovery rate correction was applied whenever more than two experimental groups were present, as detailed in [15].

## 3 Results

### 3.1 Soluble IL‐13 and CD154 at the Surface of CD4 ^+^ T Cells Increase in the Peritoneal Cavity as a Result of *H. polygyrus* Infection

**FIGURE 1:**
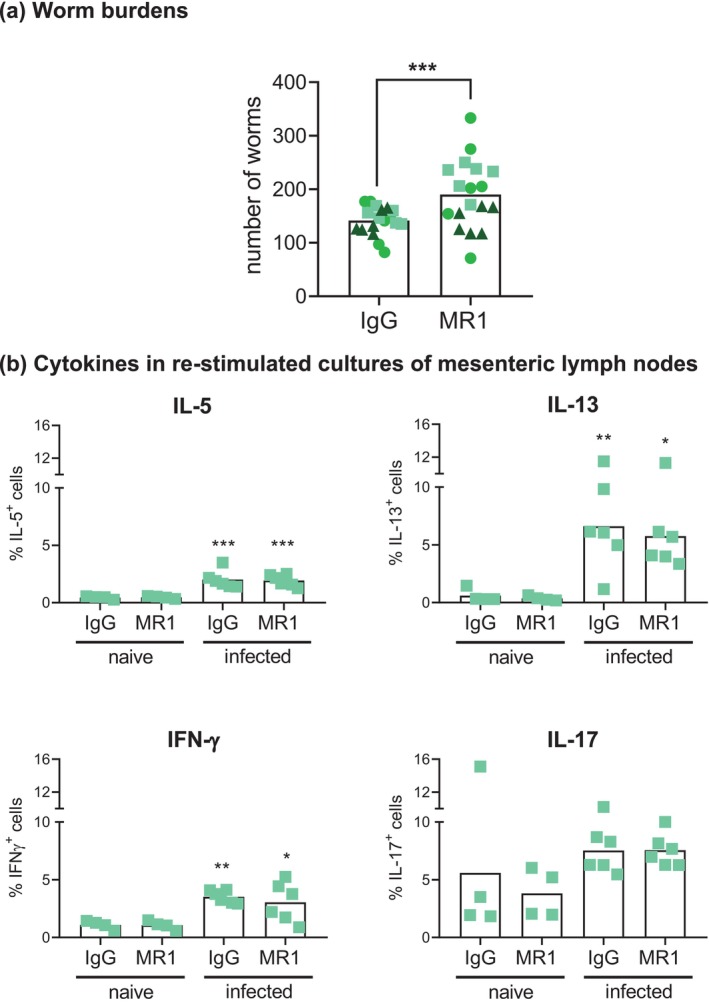
Worm burdens and mesenteric lymph node T‐cell responses in mice infected with *H. polygyrus* and treated with CD154 blocking or control antibody. Mice were infected with *H. polygyrus*, treated on day 6 with CD154 blocking antibody (MR1) or control antibody (IgG) and culled on day 9. Worm burdens in the intestine were quantified (a), and intracellular IL‐5, IL‐13, IFN‐γ and IL‐17 were measured in mesenteric lymph node CD4^+^ T cells after polyclonal ex vivo stimulation (b). Shown are individual mice and their means. Data arising from each of 3 independent experiments are presented with a different colour and symbol in (a), whereas data in (b) correspond to a single experiment. A fourth infection experiment, carried out with worm larvae from a different origin and in which CD154 was not upregulated in peritoneal cavity CD4^+^ T cells (contrary to what is shown in Figure 2), was excluded.

In this study, we evaluated the effect of blocking CD154 with the monoclonal antibody MR1 [40] on mouse infection with *H. polygyrus*. The main responses in which we were interested, namely peritoneal macrophage proliferation and polarization, have been described in this model as early as day 7 post‐infection, lasting until day 14 at least [26, 35, 37, 38, 39, 41]. We therefore blocked CD154 starting on day 6 post‐infection, culling the mice on day 9. CD154 blocking caused an increase in parasite burdens in the gut (Figure 1a). CD154 blocking did not interfere with the priming of CD4^+^ T cells, as judged from levels of intracellular cytokines in these cells retrieved from mesenteric lymph nodes and polyclonally restimulated ex vivo (Figure 1b).

**FIGURE 2:**
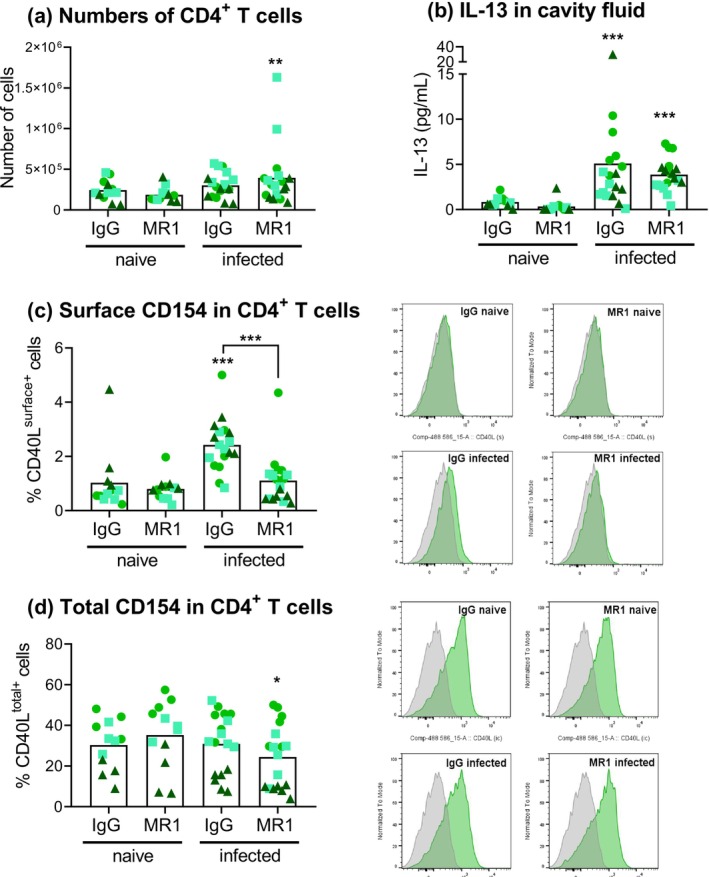
IL‐13 and CD154 are available in the peritoneal cavity of mice infected with *H. polygyrus*. Mice were treated as explained for Figure 1. Numbers of CD4^+^ T cells in the peritoneal cavity (a), concentration of IL‐13 in cavity fluid (retrieved with 1 mL of medium) (b), and cell‐surface (c) and total CD154 in CD4^+^ T cells (d) are shown. Data are presented as explained for Figure 1. Asterisks not associated with connecting lines indicate significant differences with respect to the uninfected (naïve) controls. For CD154, representative fluorescence intensity histograms are shown in addition to percentage positive cell data; FMO controls included were carried out separately for naïve and infected mice. Statistical analysis applied to the MFI data indicated that CD154 levels in control infected IgG‐treated mice were higher than those in naïve IgG‐treated mice and in infected MR1‐treated mice (*p* < 0.001 for both comparisons).

We focused on CD4^+^ T cells as sources of CD154, as this is the only cell type in the peritoneal cavity that expresses detectable CD154, both under basal conditions and after injection of IL‐4 [15]. The number of CD4^+^ T cells in the peritoneal cavity did not change appreciably in response to infection (except for a significant increase in the presence of CD154 blocking antibody that was due to two individuals with unusually high CD4^+^ T cell numbers) (Figure 2a). CD4^+^ T cell numbers were not significantly altered by CD154 blockade in comparison to the control treatment, in naïve or in infected animals.

The peritoneal cavity of mice infected with *H. polygyrus* is known to accumulate CD4^+^ T cells capable of producing type 2 cytokines and macrophages that have responded to these cytokines [26, 34, 36, 37], but direct evidence of available type 2 cytokines in the cavity is lacking. Although we could not reliably measure IL‐4, we found IL‐13 in the cavity fluid to be strongly induced by infection and not to be affected by CD154 blocking (Figure 2b).

We previously showed that CD154 constitutively expressed by naive CD4^+^ T cells is not required for peritoneal macrophages to respond to IL‐4 [15]. The focus of the present work was therefore the impact on macrophage type 2 responses of CD154 expressed by CD4^+^ T cells activated by antigen. CD154 expression in CD4^+^ T cells was significantly higher in infected than in naïve mice, in terms of percentage of positive cells as well as of fluorescent signal intensity (Figure 2c), suggestive of antigen presentation. The increase in surface CD154 was not observed in the presence of the blocking antibody, possibly because this antibody blocked the interaction with the detection antibody (which corresponds to the same clone). This suggests that CD154 blocking in vivo by the antibody administered on day 6 post‐infection lasted until day 9. Total (cell‐surface and intracellular) CD154 was not increased by infection (Figure 2d). This is expected, as antigen‐experienced CD4^+^ T cells, present even in the steady state peritoneal cavity, contain abundant intracellular CD154 [15]. In agreement with our previous results [15], non‐CD4^+^ T cells in the cavity did not express CD154, in naïve or infected animals (Figure S2).

The data thus far demonstrated that CD4^+^ T cells present in the peritoneal cavity externalise CD154 in response to *H. polygyrus* infection, suggesting local antigenic stimulation. In addition, infection induces the release of IL‐13 into the cavity, which we reason arises at least in part from Th2 cells, since TCR triggering is known to elicit type 2 cytokines from the Th2 cells accumulating in the peritoneal cavity of *H. polygyrus*‐infected mice [42].

### 3.2 CD154 Restricts LCM Proliferation in *H. polygyrus* Infection

Peritoneal cavity macrophages can be divided into three main subsets: F4/80^Hi^ MHCII^Lo^ large cavity macrophages (LCM), F4/80^Int^ MHCII^Int^ converting cavity macrophages (CCM) and F4/80^Lo^ MHCII^Hi^ small cavity macrophages (SCM) (Figure S1) [43]. LCM are tissue‐resident macrophages. The CCM and SCM in infected mice are mostly cells recruited recently in response to inflammatory signals, whereas SCM and CCM in naïve mice are cells recruited by homeostatic mechanisms and thus in some aspects different from their inflammatory counterparts [44]. Notably the SCM gate includes CD11b^+^ DC [44].

**FIGURE 3:**
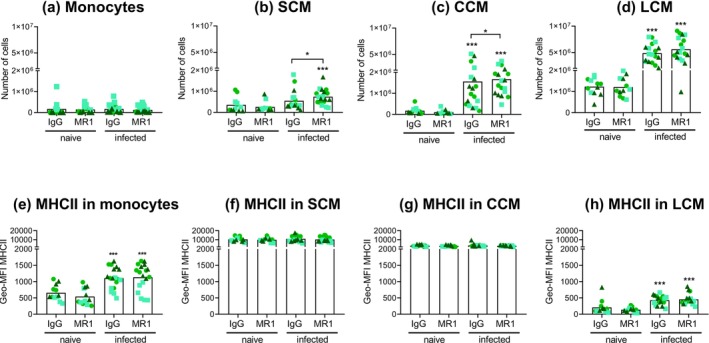
CD154 blocking potentiates the numbers of small and converting peritoneal cavity macrophages in mice infected with *H. polygyrus*. Mice were treated as explained for Figure 1 and the numbers of monocytes (a), SCM (b), CCM (c), and LCM (d) quantified and their expression of MHCII was measured (e–h). The data are presented as detailed for Figure 1. Asterisks not associated with connecting lines indicate significant differences with respect to the uninfected (naïve) controls.

At day 9 post‐infection, *H. polygyrus* infection increased the numbers of SCM, CCM and LCM in the peritoneal cavity, but did not alter the monocyte numbers (Figure 3a–d). This is broadly consistent with previous reports [35, 37, 38], but it constitutes the first description of CCM in the *H. polygyrus* model, previously described in the pleural cavity of mice infected with the filarial nematode *Litomosoides sigmodontis* [43]. CD154 blocking caused marginal further increases in mean CCM and SCM numbers, without effect on monocytes or LCM.

Basal MHCII expression was highest in SCM followed by CCM, being low in monocytes and negligible in LCM, as expected [44] (Figure 3e–h). Upon infection, monocytes and LCM displayed modest increases in MHCII expression, whereas SCM and CCM remained unchanged. Thus, all monocyte–macrophage types analysed had at least some potential to engage in specific interactions with CD4^+^ T cells. CD154 blocking did not alter MHCII expression (Figure 3e–h).

**FIGURE 4:**
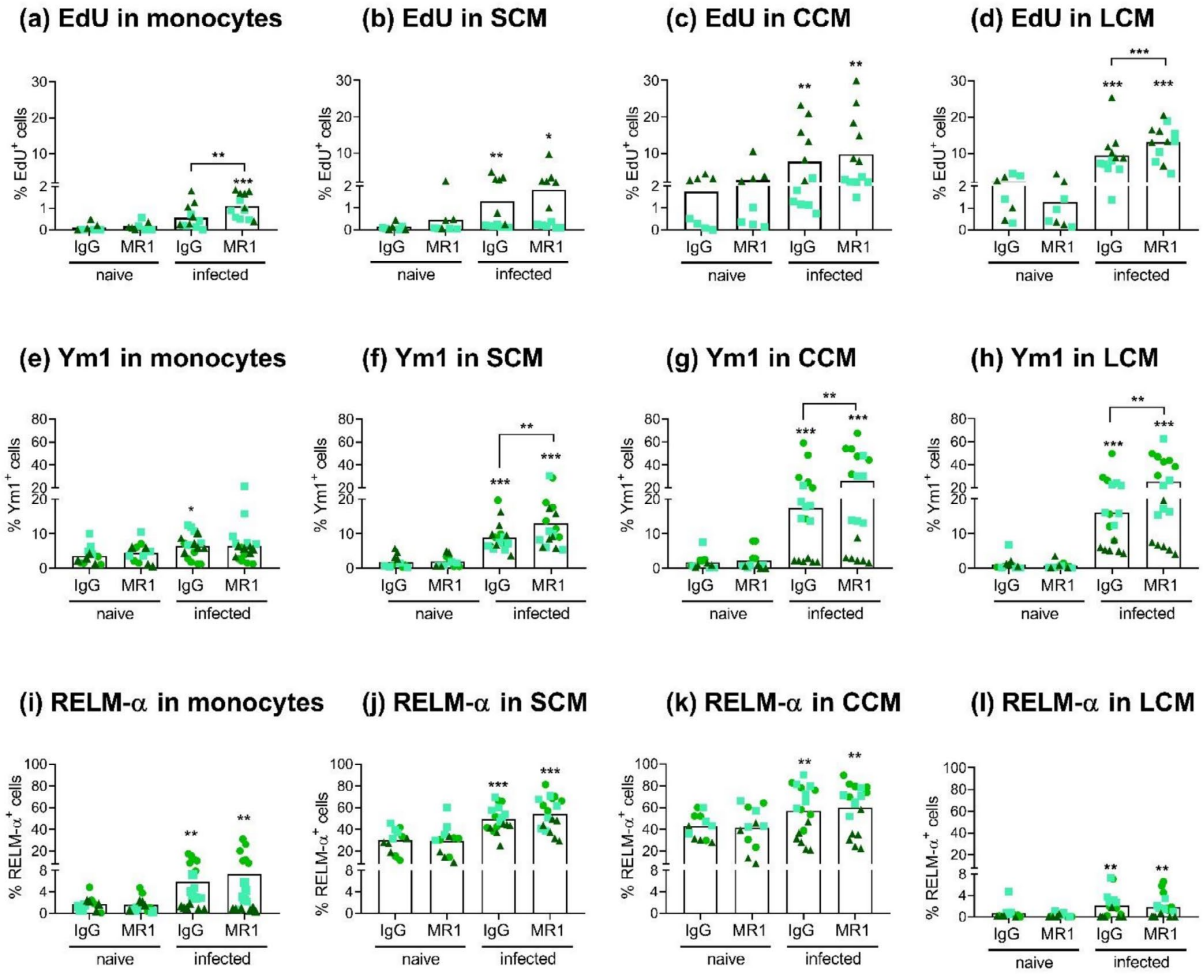
CD154 blocking potentiates aspects of the proliferation and polarization of peritoneal macrophages in *H. polygyrus* infection. Mice were treated as explained for Figure 1 and incorporation of EdU (a–d) as an indication of proliferation, and expression of Ym1 (e–h) and RELM‐α (i–l) measured in monocytes, SCM, CCM and LCM. EdU incorporation was measured only in 2 out of the 3 experiments. The data are presented as detailed for Figure 1. Asterisks not associated with connecting lines indicate significant differences with respect to the uninfected (naïve) controls.

Broadly consistent with previous reports [26, 37], infection‐induced cell proliferation in the three macrophage subsets analysed but not in monocytes (in mice treated with control antibody) (Figure 4a–d and Figure S3). Proliferation in terms of EdU incorporation was strongest in LCM followed by CCM and then SCM (Figure 4a–d). CD154 blocking further enhanced the infection‐induced proliferation of LCM both in terms of EdU incorporation (Figure 4d) and percentage of Ki‐67^Hi^ cells (Figure S3e). Ki‐67^Hi^ expression reflects cells in S and G2/M phases of the cell cycle, in contrast to total Ki‐67^+^ cells, which includes cells in the G1 phase and can include cells not engaged in proliferation [26, 45, 46]. Nonetheless, CD154 blocking during infection also resulted in a significant increase in Ki‐67^+^ LCM in comparison to non‐infected mice (Figure S3d). CD154 blocking did not alter the proliferation of SCM or CCM (Figure 4b,c and Figure S3b,c). CD154 blocking allowed monocytes to proliferate in response to infection, although to a small extent (Figure 4a and Figure S3a).

Thus, during *H. polygyrus* infection, blocking CD154 boosts the proliferation of LCM; however, at the studied time point, this did not manifest in higher LCM numbers. On the other hand, blocking CD154 boosts CCM and SCM numbers slightly, without an impact on their proliferation.

### 3.3 CD154 Restricts the M(IL‐4) Polarisation of Peritoneal Macrophages in *H. polygyrus* Infection

Two of the most widely used M(IL‐4) markers for serous cavity macrophages are Ym1 (*Chil3*) and RELM‐α [15, 25, 26, 43]. Infection induced the expression of Ym1 on all the monocyte–macrophage populations analysed (Figure 4e–h). Blocking CD154 further increased Ym1 expression in the 3 macrophage populations analysed, but not in monocytes.

Infection induced the expression of RELM‐α on all the monocyte–macrophage populations analysed (Figure 4i–l). However, this induction was extremely weak in LCM, in line with a previous report in the same mouse strain [38]. Also broadly consistent with previous reports [44, 47, 48, 49], the cells defined as SCM and CCM expressed RELM‐α in naïve mice (Figure 4j,k). Such constitutive RELM‐α expression is absent from inflammatory macrophages [44, 47, 48, 49], which likely make up the majority of the SCM and CCM present in the infection setting. Thus, infection‐induced RELM‐α expression in SCM and CCM is most probably stronger than suggested by the comparison between naïve and infected mice in Figure 4j,k. CD154 blocking did not affect RELM‐α expression in the absence of infection (consistent with our previous report [15]). It also did not alter infection‐induced RELM‐α expression by any of the cell types analysed.

In sum, during *H. polygyrus* infection, CD154 restricts Ym1 expression both by inflammatory and resident‐type macrophages but does not affect the expression of RELM‐α.

### 3.4 CD154 May Be Necessary for the Efficient Acquisition of a Resident Phenotype by Macrophages Recruited Into the Peritoneal Cavity in *H. polygyrus* Infection

**FIGURE 5:**
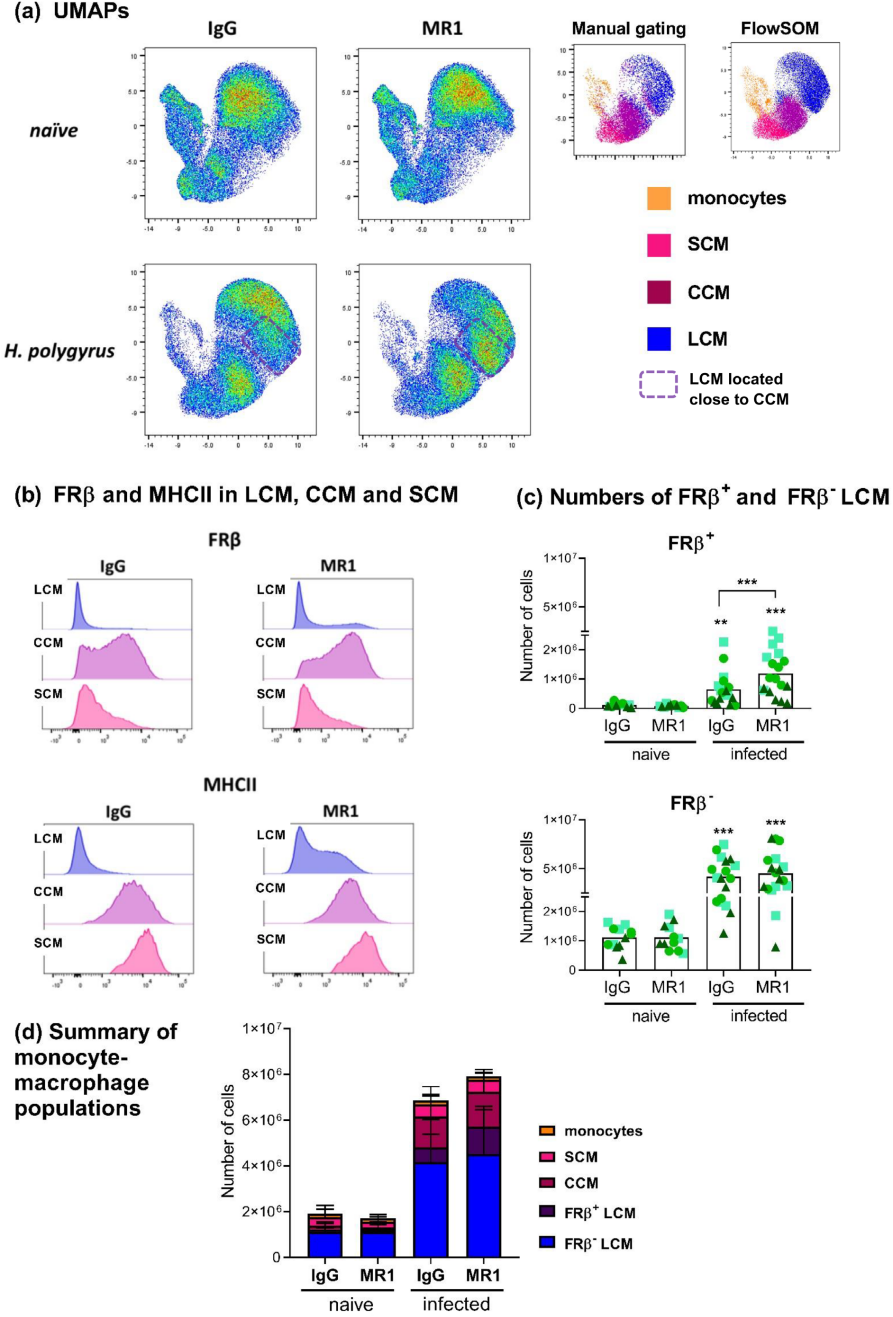
CD154 blocking appears to delay the differentiation of macrophages recruited to the peritoneal cavity into macrophages with a resident phenotype. Mice were treated as explained for Figure 1. Peritoneal cavity monocyte‐macrophages were visualised by means of UMAPs (a). Cell clusters, as defined using the conventional gating shown in Figure S1 or by means of the FlowSOM tool and identified by manual inspection, are shown side by side for a representative experiment. For infected mice, a particular sub‐population classed within LCM but located close to CCM is indicated. Representative histograms are presented for the expression of FRβ and MHCII in SCM, CCM and LCM, showing that CD154 blocking gives rise to a subset of cells classed within LCM but with anomalous expression of these two markers (b). The numbers of FRβ^+^ LCM and FRβ^−^ LCM are presented in (c), in the format detailed for Figure 1; asterisks not associated with connecting lines indicate significant differences with respect to the uninfected (naïve) controls. The mean ± SD of the numbers of monocytes, SCM, CCM, FRβ^+^ LCM and FRβ^−^ LCM in the peritoneal cavity in stacked bar graphs are presented in (d).

As mentioned, the enhancement of LCM proliferation caused by CD154 blocking was not reflected in increased LCM numbers (Figures 3d, 4d, and S3e). LCM can arise from proliferation and/or differentiation from monocytes via recruited macrophages [25]. Thus, it was conceivable that, concomitantly with boosting proliferation, CD154 blocking may decrease the numbers of LCM arising from recruited cells. To gain insights into this possibility we visualised the flow cytometry data for the monocyte–macrophage populations using UMAPs. The monocyte, SCM, CCM and LCM populations defined on the basis of conventional gating coincided with the four cell clusters defined in unsupervised form by the FlowSOM tool (Figure 5a). Inspection of the UMAPs showed that CD154 blocking in infected mice appeared to increase the frequency of cells classified as LCM but located close to CCM (Figure 5a). Upon closer inspection, at least part of these cells expressed intermediate levels of FRβ and MHCII, two markers usually associated with CCM but not with LCM [43, 44] (Figure S4). Indeed, CD154 blocking gave rise to cells classed within LCM (and having F4/80 and CD102 levels typical of LCM or even higher; Figure S5a,b) but expressing FRβ and MHCII at levels similar to those of most CCM (Figure 5b). We reason that these FRβ^+^ MHCII^+^ F4/80^Hi^ CD102^Hi^ cells are intermediate between CCM and *bona‐fide* LCM. However, we chose to call them “FRβ^+^ LCM” as they were classed by the FlowSOM tool together with LCM.

FRβ^+^ LCM tended to proliferate less strongly in response to infection than conventional FRβ^−^ LCM, this trend reaching significance in the presence of CD154 blocking antibody (Figure S6a). Thus, in terms of proliferation FRβ^+^ LCM may resemble CCM, which proliferate slightly less than bulk LCM (see Figure 4c,d). FRβ^+^ LCM did not differ from FRβ^−^ LCM in terms of infection‐induced Ym1 expression (Figure S5b), a response which is similar in CCM and bulk LCM (see Figure 4g,h). A significantly higher fraction of the FRβ^+^ LCM expressed RELM‐α in response to infection in comparison to FRβ^−^ LCM. Thus, in this respect FRβ^+^ LCM also appear as intermediate between CCM and conventional LCM (Figure S6c; Figure 4k,l).

Quantification confirmed that CD154 blocking increased the numbers of FRβ^+^ LCM, but not those of the conventional FRβ^−^ LCM (Figure 5c). Integrating this analysis with the data previously shown on SCM and CCM numbers (Figure 3b,c) strongly suggested that CD154 blocking during infection delays progress along the differentiation pathway from SCM, through CCM, to fully resident LCM. Overall, CD154 blocking during infection resulted in higher numbers of macrophages in the cavity fuelled by increases in CCM and FRβ^+^ LCM. Taken together, these two intermediate cell types increase by 35% in absolute terms in response to CD154 blocking (Figure S7). In contrast, the numbers of bulk ‐ LCM (which are dominated by FRβ^−^ LCM; Fig. 5c, d) as mentioned do not increase, probably because enhanced proliferation of LCM (Figure 4d and Figure S3e) is balanced by their reduced differentiation from recruited cells.

Taken together, these data indicate that in *H. polygyrus* infection CD154 collaborates towards the differentiation of macrophages recruited to the peritoneal cavity into LCM with a fully resident phenotype.

## 4 Discussion

The main conclusion of our work is that endogenous CD154 available in a helminth infection context can limit peritoneal macrophage responses to type 2 cytokines. This confirms our previous observations using exogenous IL‐4 together with soluble CD40 agonists or CD154 induced by antigen in TCR‐transgenic CD4^+^ T cells [15]. We previously found no evidence that blocking CD154 available under basal conditions altered peritoneal macrophage responses [15]. We therefore ascribe the effects of CD154 blocking on macrophages observed in this work (Figures 3, 4, 5) to the blocking of CD154 induced in CD4^+^ T cells by the presentation of worm antigens. This is backed by the observation that *H. polygyrus* infection by day 9 caused a significant increase in cell‐surface CD154 in CD4^+^ T cells (Figure 2c). *H. polygyrus* antigens could reach the peritoneal cavity in cell‐free form, as reported for certain intestinal bacterial antigens [50, 51], or within DCs that migrate from the gut (as documented for secondary infections with this worm [52]). Antigen presentation in the peritoneal cavity may explain at least part of the availability of type 2 cytokines in the cavity evidenced by our IL‐13 measurements (Figure 2b) and, indirectly, by macrophage polarisation and proliferation (Figure 4) [26, 37, 38, 39].

Our data do not indicate whether peritoneal macrophages in *H. polygyrus* infection are impacted by CD154 directly, indirectly, or in both manners. Our results using exogenous IL‐4 indicate that CD40 agonists can restrict M(IL‐4) polarization through direct signalling to macrophages (both via CD40 and independently of this receptor), without ruling out additional indirect effects [15]. In the infection context, a direct CD154 signal to macrophages could occur in the context of antigen‐specific interactions with CD4^+^ T cells, as all macrophage types analysed express MHCII (Figure 3e–h). However, most LCM express only low levels of MHCII in comparison with inflammatory macrophages (Figures 3f–h and 5b) and in all likelihood DCs. The DCs that enter the peritoneal cavity in secondary *H. polygyrus* infection become activated [52], which indicates that they present antigen in that context. It should therefore not be ruled out that LCM, or even peritoneal macrophages altogether, are dispensable for the antigen‐specific interactions that cause the upregulation of cell‐surface CD154 observed in cavity CD4^+^ T cells in primary infection. CD154 induced in CD4^+^ T cells by antigen‐specific interactions with DCs and/or macrophage types expressing high levels of MHCII (e.g., SCM) could still impact directly on those macrophages that express less MHCII (e.g., LCM). Such bystander cell CD40‐CD154 interactions are documented for DCs during immune priming in lymph nodes [53]. Also, macrophages interact via CD40 with CD154 on CD4^+^ T cells in the basal peritoneal cavity, that is, without the need for antigen presentation [15]. Macrophages in the *H. polygyrus* infection context can conceivably also be influenced by CD154 indirectly, that is, via soluble mediators secreted by other APCs in response to CD40 ligation. IFN‐γ, known to restrict M(IL‐4) polarization and macrophage proliferation in response to M‐CSF [54, 55, 56], is a probable such mediator. C57BL/6 mount a mixed type 1–type 2 response to *H. polygyrus* infection [38], and the well‐known CD40–IL‐12–IFN‐γ axis can be potentiated by IL‐4 [57, 58, 59, 60].

CD154 blocking significantly enhanced macrophage proliferation in LCM but not in inflammatory peritoneal macrophages during *H. polygyrus* infection (Figure 4a–d). This is in line with LCM expressing more CD40 and being more sensitive to CD154‐mediated inhibition of M(IL‐4) polarization than inflammatory macrophages [15], and may suggest that a direct effect of CD154 on macrophages is at play. The effect of CD154 on Ym1 but not RELM‐α expression in CCM and SCM contrasts with our previous observations, using exogenous IL‐4, that RELM‐α expression is generally more sensitive to blunting by CD154 than Ym1 expression [15]. This may be explained by the experimental systems differing in: (i) the set of stimuli inducing RELM‐α and Ym1, as these markers can be induced independently of IL‐4 even in helminth models [61, 62], and/or (ii) the relative weight of direct vs indirect effects of CD154 on macrophages, previously discussed.

CD154 appears to promote the transition from recruited macrophages into fully resident cells in the peritoneal cavity (Figures 3b,c and 5). Similar to what was previously discussed, this effect could be due to macrophages sensing CD154 in the context of antigen‐specific interactions or as bystander cells, and/or to mediators released by other cell types in response to CD154. The recruited‐to‐resident macrophage differentiation process has been well studied for the pleural cavity, in the context of infection with *L. sigmodontis* [43]. In the filarial model, macrophage tissue residency takes place efficiently in C57BL/6 but not in BALB/c mice, which accumulate SCM and CCM [43]. Interestingly, the LCM found in the pleural cavity of *L. sigmodontis*‐infected BALB/c mice are heterogeneous for expression of FRβ and MHCII (whereas those in C57BL/6 mice are negative) even if they have uniformly high expression of F4/80 and CD102 [43]. Thus, aspects of the macrophage dynamics in the peritoneal cavity of *H. polygyrus*‐infected C57BL/6 mice treated with CD154 blocking antibody are reminiscent of the pleural cavity of *L. sigmodontis*‐infected BALB/c mice [43].

Even in the absence of CD154 blocking, the peritoneal cavity of *H. polygyrus*‐infected C57BL/6 mice contains abundant CCM and FRβ^+^ LCM (Figures 3c and 5). In the *L. sigmodontis* model, monocyte‐to‐LCM differentiation is promoted by type 2 cytokines, more abundant in infected C57BL/6 mice, in which the response is markedly Th2, than in infected BALB/c mice, in which the response is mixed Th1/Th2 [43]. By analogy, IL‐4/IL‐13 may promote the differentiation of recruited macrophages into LCM in the peritoneal cavity and these cytokines may be limiting for this process in the mixed Th1/Th2 context of *H. polygyrus* infection in C57BL/6 mice [38].

The discussion in the previous paragraphs leads to the hypothesis that both type 2 cytokines and CD154 promote the transition from recruited macrophages into LCM. The possibility that CD154 favours macrophage acquisition of residency *through* type 2 cytokines appears remote, as TCR engagement suffices for the production of these cytokines by Th2 cells [42], and IL‐13 levels in the peritoneal cavity of infected mice were not affected by CD154 blocking (Figure 2b). The alternative possibility that type 2 cytokines act via CD154 is equally unlikely, as IL‐4 diminishes basal and antigen‐induced CD154 expression [13, 15]. Therefore, it is conceivable that type 2 cytokines and CD154 promote the acquisition of macrophage residency acting as converging signals. If this is so, residence acquisition would be the first case of a macrophage response towards which CD154 and type 2 cytokines collaborate, in contrast to the blunting by CD154 of IL‐4‐induced macrophage polarization and proliferation (Figure 4) [15].

CD154 blocking caused an increase in adult parasite burdens (Figure 1 a). Blocking was carried out between days 6 and 9 post‐infection, a time window during which the overwhelming majority of the parasites in infected C57BL/6 mice are still in the duodenal submucosa (see Figure S1 in [63]). In addition, adult worm expulsion in C57BL/6 mice only starts past 12 weeks of infection [64]. Hence, the effect of CD154 blocking is in all likelihood due to differences in larval parasite survival/development in the submucosa. Histological analyses of this stage show leukocyte infiltration and accumulation of IgE and IgG antibodies near the parasites [33, 63]. CD154 blocking as carried out in our work most probably blunts these antibody responses, which require MHCII in B cells [63] and hence must require CD40 signalling, as do class‐switched T‐cell dependent B cell responses in general [65]. Although the early IgE and IgG responses to *H. polygyrus* are dominated by a polyclonal component, the accumulation of these antibodies around the larvae [63] suggests that they may promote larval attrition in the gut tissue. C57BL/6 mice lacking mature B cells show only a trend towards higher adult parasite burdens than wild‐type mice on day 14 post‐infection [63]. However, any anti‐larval effects of antibodies may be more marked when measured on day 9 post‐infection (as done in our work), given that larval development and egress to the gut lumen continues past day 9 [63]. The above hypothesis does not exclude that CD154 may potentiate anti‐larval effector mechanisms of gut resident or recruited macrophages. While type 2‐polarised macrophages and IL‐4 are important for the expulsion of adult *H. polygyrus* [66, 67], it is possible that more conventional macrophage effector mechanisms (e.g., production of inflammatory cytokines and nitric oxide [20]) are operative against larvae at the tissue site.

## 5 Conclusions

Infection with the gut‐dwelling nematode *H. polygyrus* results in up‐regulation of surface CD154 expression on peritoneal CD4^+^ T cells, suggestive of antigen presentation at this site. This CD154, directly and/or indirectly, restricts peritoneal macrophage proliferation and expression of Ym1, in line with CD40 agonists limiting IL‐4‐induced peritoneal macrophage polarization and proliferation in reductionist systems [15]. In addition, CD154, again directly or indirectly, appears to promote the acquisition of a resident phenotype by macrophages recruited to the cavity in response to infection. Collectively, these findings suggest that in the context of a mixed Th1/Th2 response, antigen‐induced CD154 shifts the dynamics of resident macrophage accumulation away from locally derived and towards monocyte‐derived cells.

## Author Contributions

**M.S.‐M., J.P., L.Z., G.B., R.J.D.:** investigation, writing – review, editing, and revision. **B.C.:** investigation. **P.P.:** conceptualization, investigation, writing – review, editing, and revision. **C.M.F.:** conceptualization, writing – review, editing, and revision. **J.E.A.:** conceptualization, project administration/oversight, writing – review, editing, and revision. **A.D.:** conceptualization, project administration/oversight, writing – original draft.

## Funding

This work was funded by MRC‐UK MR/V011235/1 and Wellcome Trust grants 106898/A/15/Z (to J.E.A.) and by the Wolfson‐Bob Sim Uruguay Initiative, the Company of Biologists, ANII (Government of Uruguay) and PEDECIBA through travel grants to M.S.‐M.

## Disclosure

The authors have nothing to report.

## Supporting information


**Data S1:** Supporting Information.

**Figure S1:** Flow cytometry gating strategies used. The separation of non‐monocyte CD11b^+^ cells into SCM, CCM and LCM, based on F4/80 and MHCII, was also aided by the patterns of expression of CD73, FRβ and CD102, as shown in the heat maps for these three markers below the main gating strategy.
**Figure S2:** Surface and total CD154 expression in peritoneal cavity cells of naïve and *H. polygyrus*‐infected mice. CD154 was measured by flow cytometry in CD4^+^ T cells (CD4), B cells (B), monocytes (Mo), SCM, CCM, LCM, eosinophils (Eo), neutrophils (Neu) and other cells present in the peritoneal cavity of naïve (“n”) and *H. polygyrus*‐infected (“Hp”) mice. The data on CD4^+^ T cells are the same presented in Figure 2c,d. Data for eosinophils, neutrophils and “other cells” are available from a single experiment. The data are presented as explained for Figure 1.
**Figure S3:** Ki‐67 expression in peritoneal monocyte–macrophage populations of mice infected with *H. polygyrus* and treated with CD154 blocking or control antibody. Mice were treated as explained for Figure 1 and Ki‐67 expression measured in monocytes (a), SCM (b), CCM (c) and LCM (d) as an indication of cycling cells. In addition, Ki‐67^Hi^ cells (i.e., cells the G2/M phases of cell cycle specifically^61^) were quantitated in the case of LCM (e). Ki‐67^Hi^ cells could not be discriminated for monocytes, SCM and CCM.
**Figure S4:** FRβ and MHCII expression represented in heat maps on the UMAP visualisation of peritoneal monocyte–macrophages. Data are shown for infected mice only. The subpopulation of LCM located close to CCM indicated in Figure 5a is also shown.
**Figure S5:** F4/80 and CD102 expression in FRβ^+^ and FRβ^−^ LCM, and for comparison in SCM and CCM, in mice infected with *H. polygyrus* and treated with CD154 blocking or control antibody. Mice were treated as explained for Figure 1, and peritoneal cavity monocyte–macrophages analysed for F4/80 (a) and CD102 expression (b). Data, for infected mice only, are presented as explained for Figure 1.
**Figure S6:** EdU incorporation and Ym1 and RELM‐α expression in FRβ^+^ and FRβ^−^ LCM during *H. polygyrus* infection. The data from Figure 4d,h,l were re‐analysed for FRβ^−^ and FRβ^+^ LCM separately. Data are presented as explained for Figure 1.
**Figure S7:** Cell number data for CCM and FRβ^+^ LCM added together. Mice were treated as explained for Figure 1 and the cell number data shown in Figure 3c,d were re‐analysed by adding the numbers for CCM and FRβ^+^ LCM. The data are presented as detailed for Figure 1. Asterisks not associated with connecting lines indicate significance differences with respect to the uninfected (naïve) controls.

## Data Availability

The data that support the findings of this study are available on request from the corresponding author. The data are not publicly available due to privacy or ethical restrictions.
