# Supplementary material for: CD154 Restricts Helminth‐Induced Macrophage Polarisation and Proliferation While Promoting Tissue Residence
Source: Parasite Immunol. 2025 Dec 12;47(12):e70043. doi: 10.1111/pim.70043 (PMC12701298; doi:10.1111/pim.70043)
Supplement: Supplementary file 1 — Data S1: Supporting Information. [file PIM-47-e70043-s002.docx]

**Supplementary Table 1. Flow cytometry probes.**

| **Probe name** | **Dilution or concentration** | **Clone** | **Manufacturer** | **# cat** | **Secondary reagent** |
| --- | --- | --- | --- | --- | --- |
| Alexa Fluor 700 anti mouse CD19 | 1/400 | 6D5 | Biolegend | 115528 |  |
| anti mouse RELM-α | 1 µg/mL | Polyclonal Rabbit antibody | PeproTech | 500-P214 | Zenon Rabbit IgG labeling kit – AF488– Invitrogen (Thermo Fisher #Z25302) |
| anti mouse Ym1/Chil3- biotinylated | 4 µg/mL | Polyclonal Goat IgG | R&D | BAF2446 | BV421 Streptavidin (Biolegend #405225) |
| APC anti mouse FRβ | 1/400 | 10/FR2 | Biolegend | 153306 |  |
| APC-Cy7 anti mouse CD3 | 1/200 | 17A2 | Biolegend | 100222 |  |
| APC-Cy7 anti mouse I-A/I-E | 1/400 | M5/114.15.2 | Biolegend | 107628 |  |
| BUV395 anti mouse CD102 | 1/400 | 3C4(mIC2/4) | BD Bioscience | 740227 |  |
| BUV737 anti mouse Ly-6G | 1/400 | 1A8 | BD Bioscience | 741813 |  |
| BV510 anti mouse Ly-6C | 1/400 | HK1.4 | Biolegend | 128033 |  |
| BV605 anti mouse CD11c | 1/400 | N418 | Biolegend | 117334 |  |
| BV605 anti mouse CD73 | 1/400 | [TY/11.8](https://www.biolegend.com/de-at/search-results?Clone=TY/11.8) | Biolegend | 127215 |  |
| BV650 anti mouse CD4 | 1/400 | RM4-5 | Biolegend | 100555 |  |
| BV711 anti mouse CD11b | 1/400 | M1/70 | Biolegend | 101242 |  |
| BV785 anti mouse CD45 | 1/400 | 30-F11 | Biolegend | 103149 |  |
| FITC anti mouse  IL-17A | 1/100 | TC11-18H10.1 | Biolegend | 506908 |  |
| LIVE/DEAD™ Fixable Aqua | 1/250 | Not applicable | Invitrogen (Thermo Fisher) | L34957 |  |
| PE anti mouse CD154 | 1/200 | MR1 | Biolegend | 106506 |  |
| PE anti mouse/human IL-5 | 1/100 | TRFK5 | Biolegend | 504304 |  |
| PE/Dazzle™ 594 anti mouse IFN-γ | 1/100 | XMG1.2 | Biolegend | 505846 |  |
| PE-CF594 anti mouse Siglec-F | 1/400 | E50-2440 | BD Bioscience | 562757 |  |
| PE-Cy7 anti mouse F4/80 | 1/200 | BM8 | Biolegend | 123114 |  |
| PE-Cy7 anti mouse  IL-13 | 1/100 | W17010B | Biolegend | 159408 |  |

**Supplementary Figure Legends**

**Supplementary Figure 1. Flow cytometry gating strategies used.** The separation of non-monocyte CD11b^+^ cells into SCM, CCM and LCM, based on F4/80 and MHCII, was also aided by the patterns of expression of CD73, FRβ and CD102, as shown in the heat maps for these 3 markers below the main gating strategy.

**Supplementary Figure 2. Surface and total CD154 expression in peritoneal cavity cells of naïve and *H. polygyrus*-infected mice.** CD154 was measured by flow cytometry in CD4^+^ T cells (CD4), B cells (B), monocytes (Mo), SCM, CCM, LCM, eosinophils (Eo), neutrophils (Neu) and other cells present in the peritoneal cavity of naïve (“n”) and *H. polygyrus*-infected (“Hp”) mice. The data on CD4^+^ T cells are the same presented in Figure 2 c, d. Data for eosinophils, neutrophils and “other cells” are available from a single experiment. The data are presented as explained for Figure 1.

**Supplementary Figure 3. Ki-67 expression in peritoneal monocyte-macrophage populations of mice infected with *H. polygyrus* and treated with CD154 blocking or control antibody.** Mice were treated as explained for Figure 1 and Ki-67 expression measured in monocytes (a), SCM (b), CCM (c) and LCM (d) as an indication of cycling cells. In addition, Ki-67^Hi^ cells (i.e. cells the G2/M phases of cell cycle specifically ^62^) were quantitated in the case of LCM (e). Ki-67^Hi^ cells could not be discriminated for monocytes, SCM and CCM.

**Supplementary Figure 4. FRβ and MHCII expression represented in heat maps on the UMAP visualization of peritoneal monocyte-macrophages.** Data are shown for infected mice only. The subpopulation of LCM located close to CCM indicated in Figure 5 a is also shown.

**Supplementary Figure 5. F4/80 and CD102 expression in FRβ^+^ and FRβ^-^ LCM, and for comparison in SCM and CCM, in mice infected with *H. polygyrus* and treated with CD154 blocking or control antibody.** Mice were treated as explained for Figure 1, and peritoneal cavity monocyte-macrophages analyzed for F4/80 (a) and CD102 expression (b). Data, for infected mice only, are presented as explained for Figure 1.

**Supplementary Figure 6. EdU incorporation and Ym1 and RELM-α expression** **in FRβ^+^ and FRβ^-^ LCM during *H. polygyrus* infection.** The data from Figure 4 d, h and l were re-analyzed for FRβ^-^ and FRβ^+^ LCM separately. Data are presented as explained for Figure 1.

**Supplementary Figure 7. Cell number data for CCM and FRβ^+^ LCM added together.** Mice were treated as explained for Figure 1 and the cell number data shown in Figure 3 c, d were re-analyzed by adding the numbers for CCM and FRβ^+^ LCM. The data are presented as detailed for Figure 1. Asterisks not associated with connecting lines indicate significance differences with respect to the uninfected (naïve) controls.
