# Supplementary material for: CD154 Restricts Helminth‐Induced Macrophage Polarisation and Proliferation While Promoting Tissue Residence
Source: Parasite Immunol. 2025 Dec 12;47(12):e70043. doi: 10.1111/pim.70043 (PMC12701298; doi:10.1111/pim.70043)
Supplement: Supplementary file 2 — Figure S1: Flow cytometry gating strategies used. The separation of non‐monocyte CD11b+ cells into SCM, CCM and LCM, based on F4/80 and MHCII, was also aided by the patterns of expression of CD73, FRβ and CD102, as shown in the heat maps for these three markers below the main gating strategy. Figure S2: Surface and total CD154 expression in peritoneal cavity cells of naïve and H. polygyrus‐infected mice. CD154 was measured by flow cytometry in CD4+ T cells (CD4), B cells (B), monocytes (Mo), SCM, CCM, LCM, eosinophils (Eo), neutrophils (Neu) and other cells present in the peritoneal cavity of naïve (“n”) and H. polygyrus‐infected (“Hp”) mice. The data on CD4+ T cells are the same presented in Figure 2c,d. Data for eosinophils, neutrophils and “other cells” are available from a single experiment. The data are presented as explained for Figure 1. Figure S3: Ki‐67 expression in peritoneal monocyte–macrophage populations of mice infected with H. polygyrus and treated with CD154 blocking or control antibody. Mice were treated as explained for Figure 1 and Ki‐67 expression measured in monocytes (a), SCM (b), CCM (c) and LCM (d) as an indication of cycling cells. In addition, Ki‐67Hi cells (i.e., cells the G2/M phases of cell cycle specifically61) were quantitated in the case of LCM (e). Ki‐67Hi cells could not be discriminated for monocytes, SCM and CCM. Figure S4: FRβ and MHCII expression represented in heat maps on the UMAP visualisation of peritoneal monocyte–macrophages. Data are shown for infected mice only. The subpopulation of LCM located close to CCM indicated in Figure 5a is also shown. Figure S5: F4/80 and CD102 expression in FRβ+ and FRβ− LCM, and for comparison in SCM and CCM, in mice infected with H. polygyrus and treated with CD154 blocking or control antibody. Mice were treated as explained for Figure 1, and peritoneal cavity monocyte–macrophages analysed for F4/80 (a) and CD102 expression (b). Data, for infected mice only, are presented as explained for Fi [file PIM-47-e70043-s001.pptx]

## Slide 1
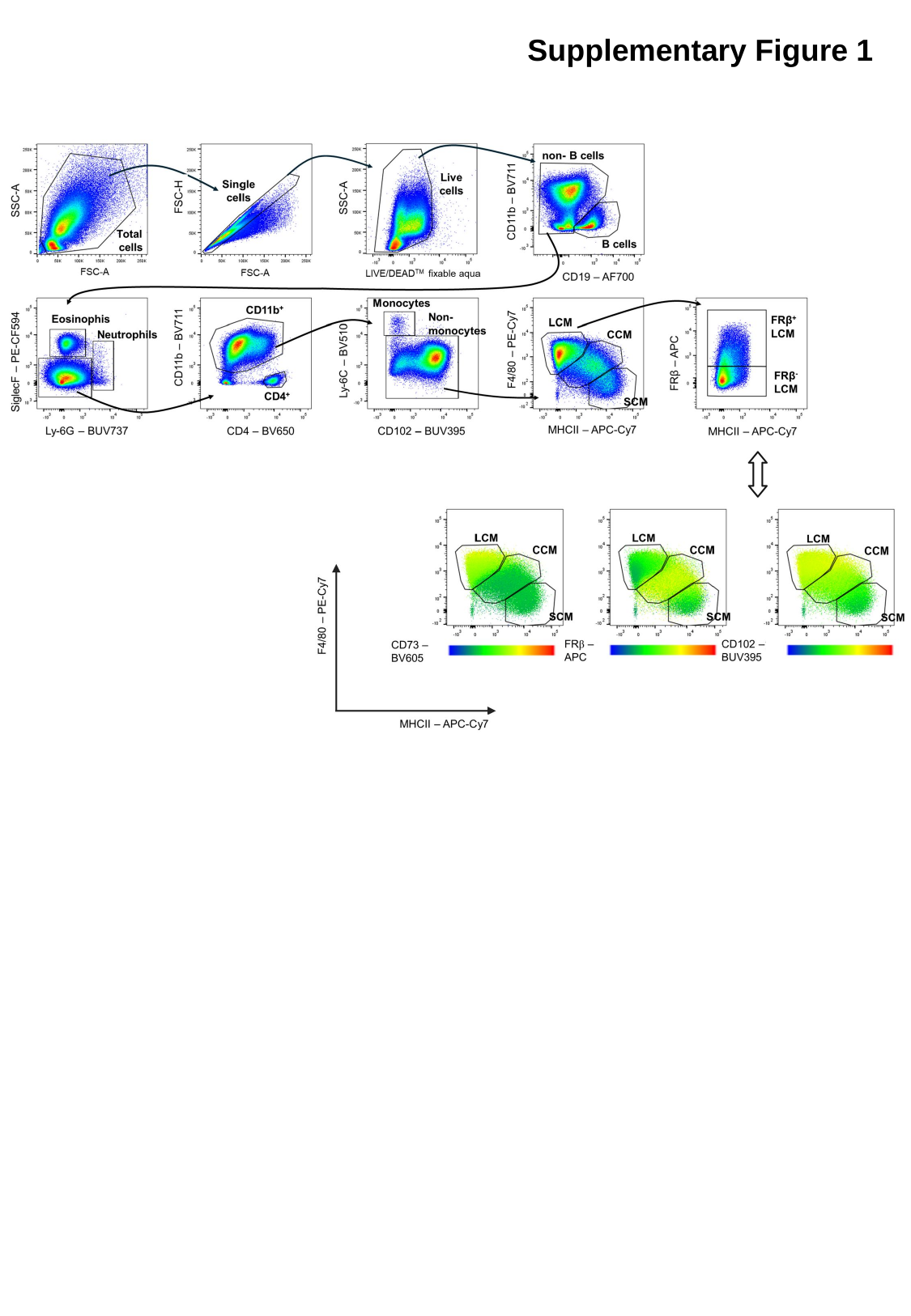

Supplementary Figure 1

## Slide 2
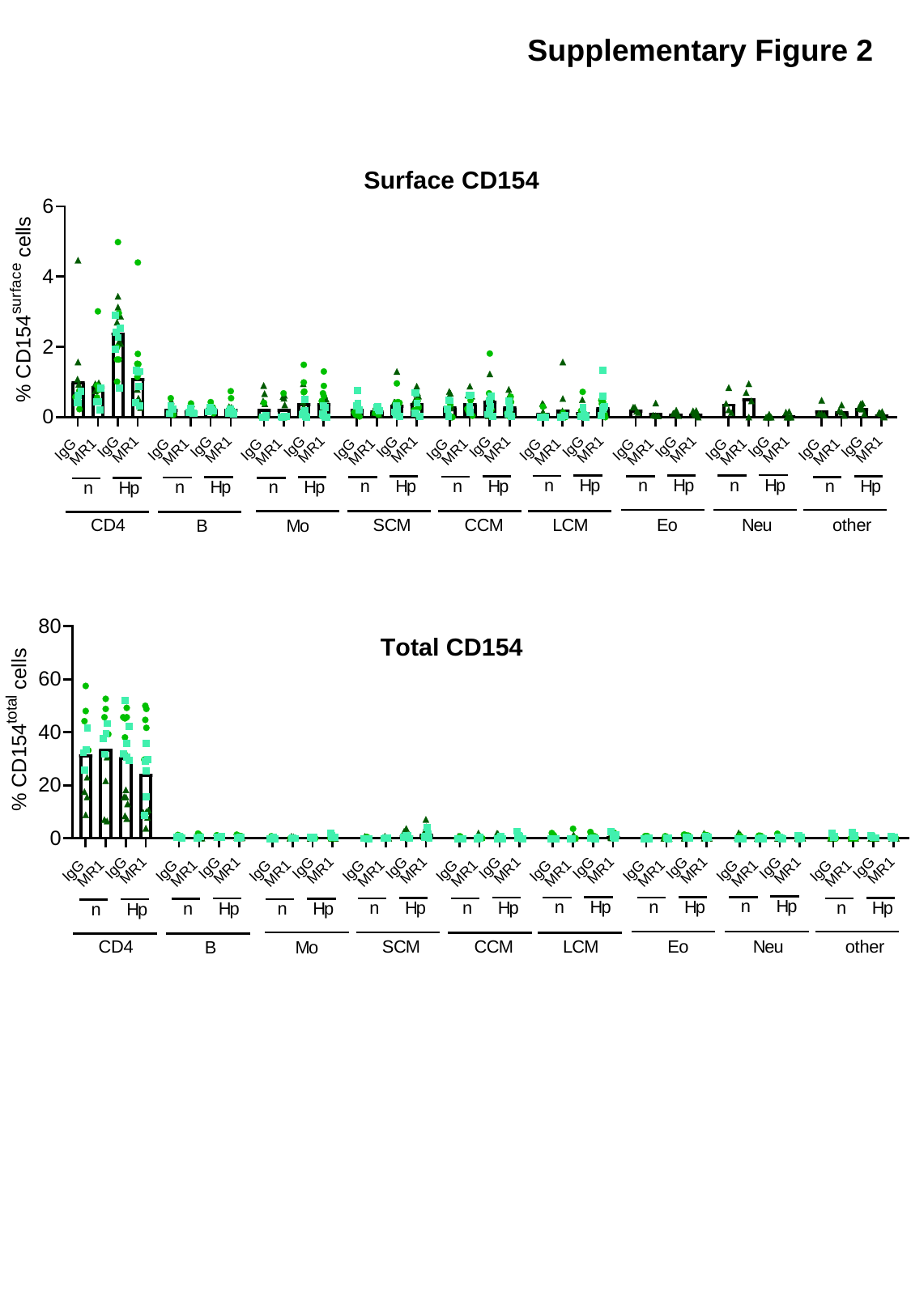

Supplementary Figure 2

## Slide 3
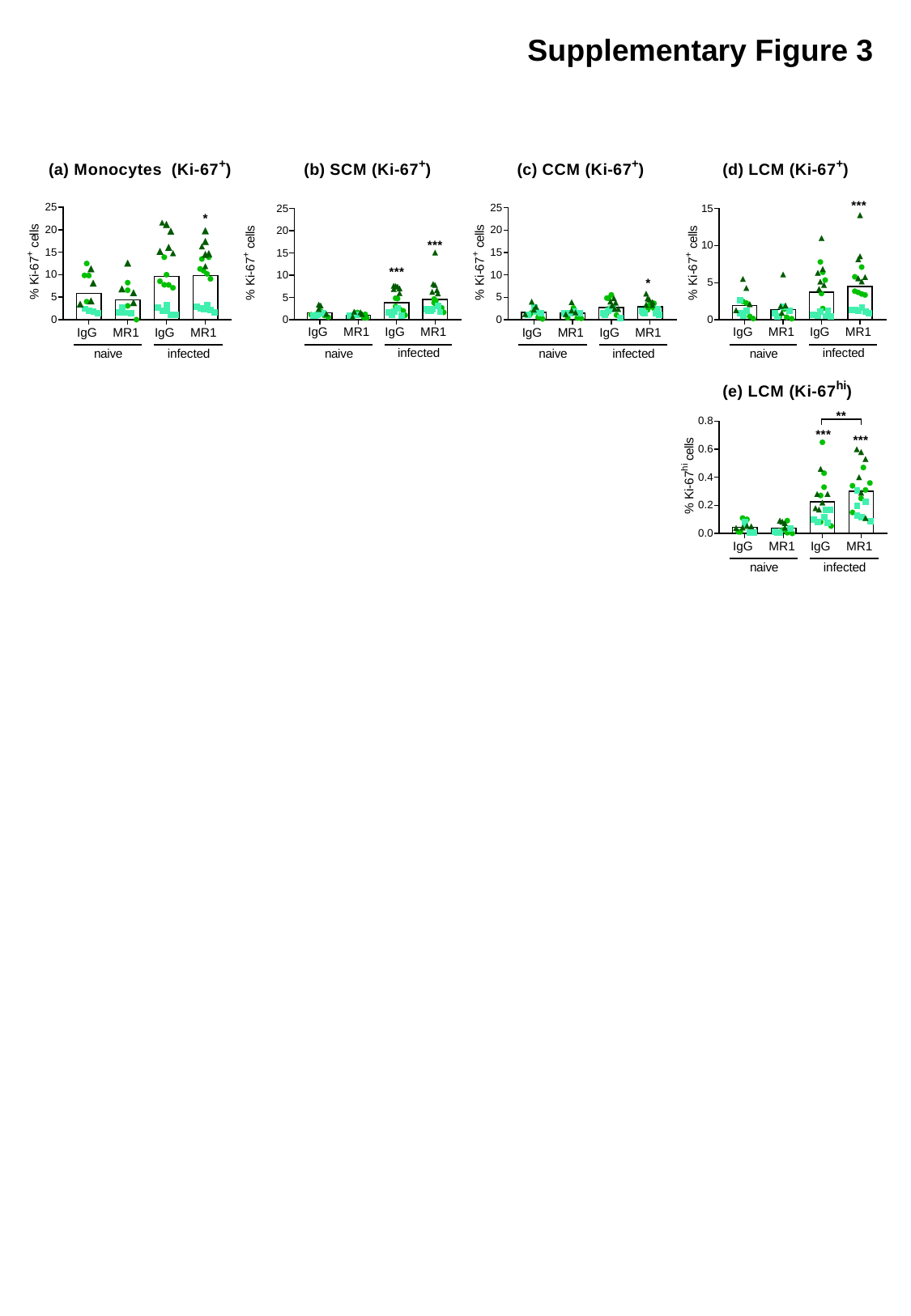

Supplementary Figure 3

## Slide 4
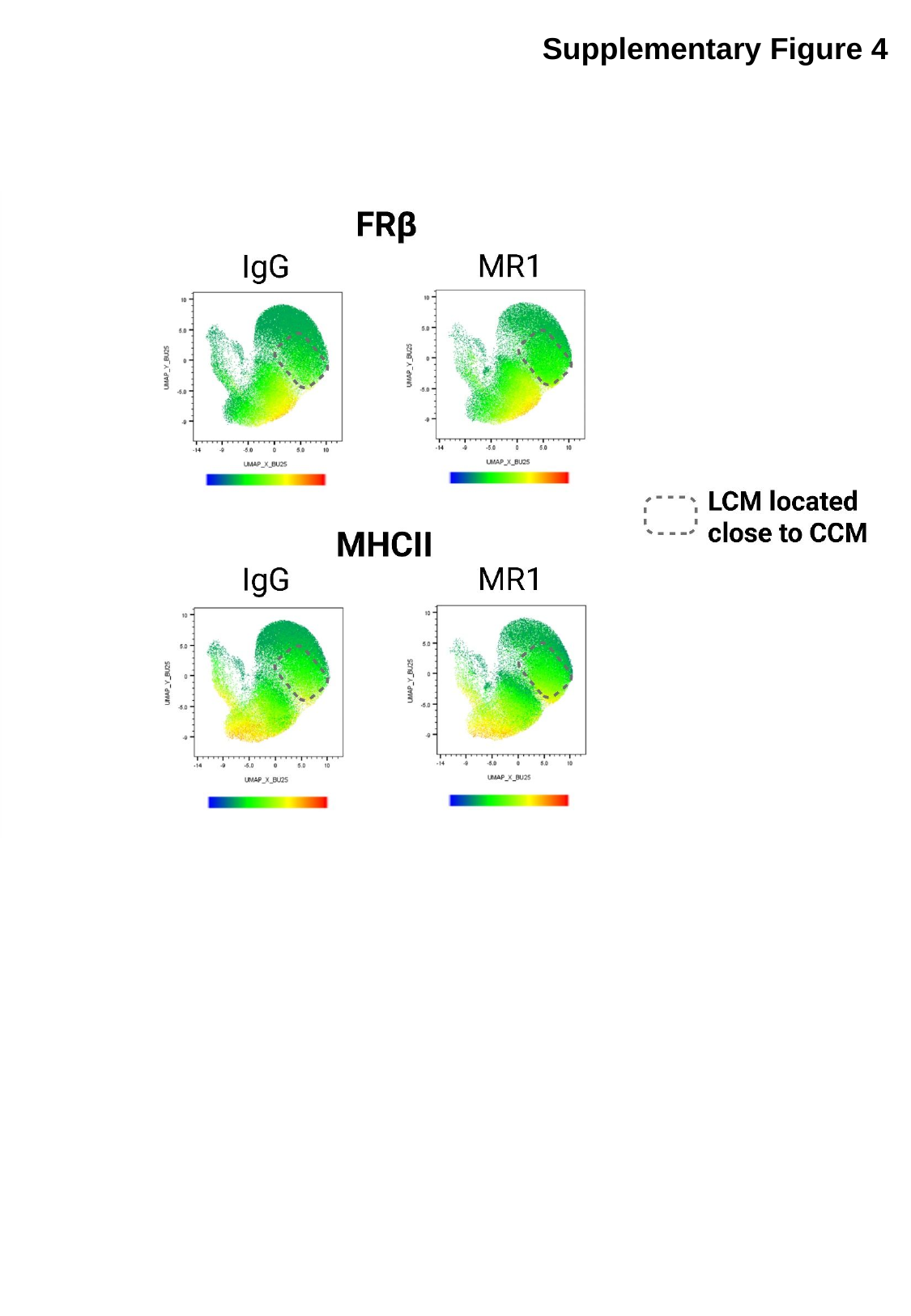

Supplementary Figure 4

## Slide 5
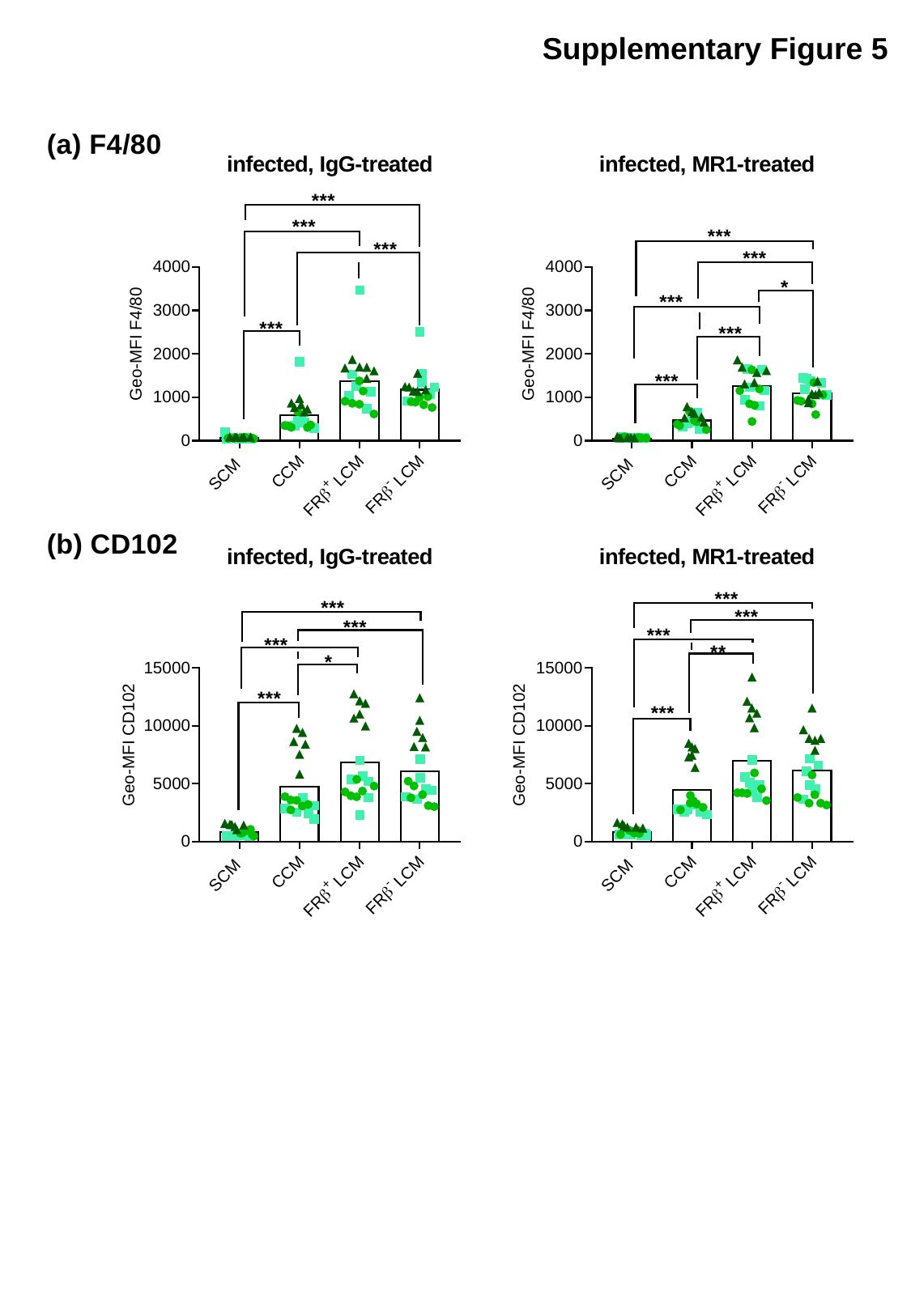

Supplementary Figure 5

## Slide 6
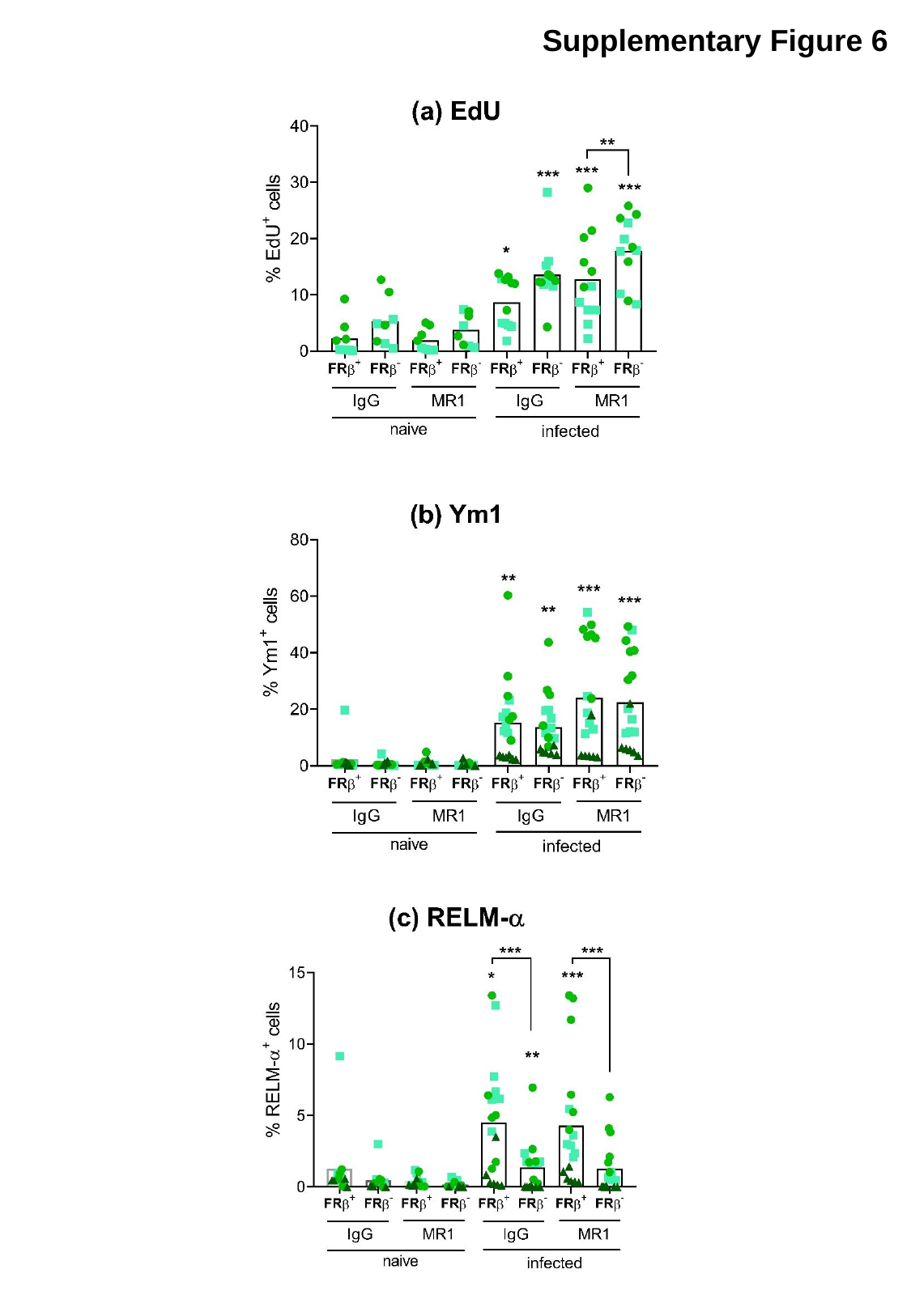

Supplementary Figure 6

## Slide 7
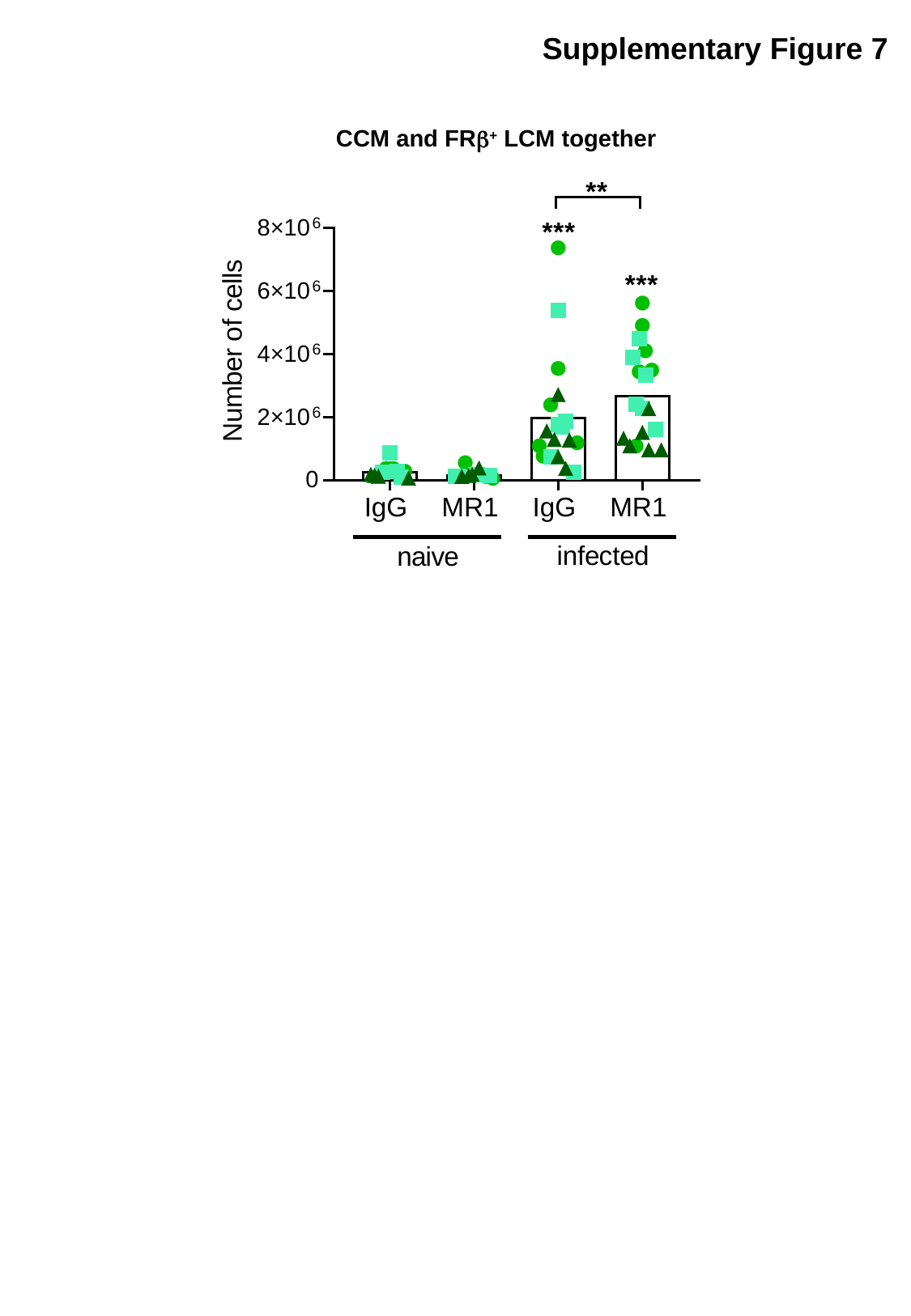

Supplementary Figure 7
CCM and FRb+ LCM together
